# Low availability of functional seed trait data from the tropics could negatively affect global macroecological studies, predictive models and plant conservation

**DOI:** 10.1093/aob/mcac130

**Published:** 2022-11-09

**Authors:** Anne M Visscher, Filip Vandelook, Eduardo Fernández-Pascual, Laura Victoria Pérez-Martínez, Tiziana Ulian, Mauricio Diazgranados, Efisio Mattana

**Affiliations:** Royal Botanic Gardens, Kew, Wakehurst, Ardingly, Haywards Heath, West Sussex, RH17 6TN, UK; Meise Botanic Garden, Nieuwelaan 38, 1860 Meise, Belgium; IMIB – Biodiversity Research Institute, University of Oviedo, Mieres, Spain; Royal Botanic Gardens, Kew, Wakehurst, Ardingly, Haywards Heath, West Sussex, RH17 6TN, UK; Hawkesbury Institute for the Environment, Western Sydney University, Richmond, NSW 2753, Australia; Royal Botanic Gardens, Kew, Wakehurst, Ardingly, Haywards Heath, West Sussex, RH17 6TN, UK; Royal Botanic Gardens, Kew, Wakehurst, Ardingly, Haywards Heath, West Sussex, RH17 6TN, UK; Royal Botanic Gardens, Kew, Wakehurst, Ardingly, Haywards Heath, West Sussex, RH17 6TN, UK

**Keywords:** Alpine germination, desiccation sensitivity, functional seed traits, geographical bias, global ecological studies, páramo ecosystems, post-dispersal embryo growth, relative embryo size, tropical plant diversity

## Abstract

**Background:**

Plant seeds have many traits that influence ecological functions, *ex situ* conservation, restoration success and their sustainable use. Several seed traits are known to vary significantly between tropical and temperate regions. Here we present three additional traits for which existing data indicate differences between geographical zones. We discuss evidence for geographical bias in availability of data for these traits, as well as the negative consequences of this bias.

**Scope:**

We reviewed the literature on seed desiccation sensitivity studies that compare predictive models to experimental data and show how a lack of data on populations and species from tropical regions could reduce the predictive power of global models. In addition, we compiled existing data on relative embryo size and post-dispersal embryo growth and found that relative embryo size was significantly larger, and embryo growth limited, in tropical species. The available data showed strong biases towards non-tropical species and certain families, indicating that these biases need to be corrected to perform truly global analyses. Furthermore, we argue that the low number of seed germination studies on tropical high-mountain species makes it difficult to compare across geographical regions and predict the effects of climate change in these highly specialized tropical ecosystems. In particular, we show that seed traits of geographically restricted páramo species have been studied less than those of more widely distributed species, with most publications unavailable in English or in the peer-reviewed literature.

**Conclusions:**

The low availability of functional seed trait data from populations and species in the tropics can have negative consequences for macroecological studies, predictive models and their application to plant conservation. We propose that global analyses of seed traits with evidence for geographical variation prioritize generation of new data from tropical regions as well as multi-lingual searches of both the grey- and peer-reviewed literature in order to fill geographical and taxonomic gaps.

## INTRODUCTION

**Fig. 1.**
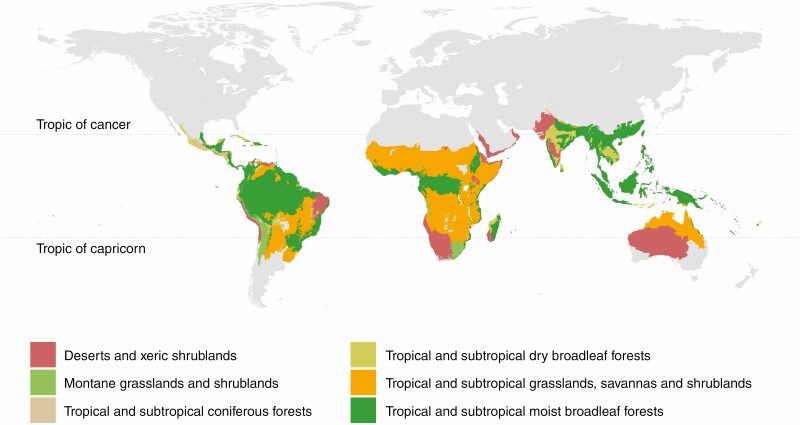
Tropical biomes of the world. Major tropical biomes as defined by the terrestrial regionalization of the World Wildlife Fund (Olson *et al.*, 2001).

Recent reports have highlighted the existence of geographical biases in ecological studies and global reviews. For example, Culumber *et al.* (2019) detected geographical bias in seven out of nine topics covering broad ecological and evolutionary research, with the tropics (Fig. 1) being significantly under-represented, particularly for studies on plants. In addition, Feeley *et al.* (2017) pointed out that, whilst about a third of the Earth’s land surface lies within the tropics and the vast majority of Earth’s species are tropical, tropical ecosystems are essentially absent from nearly all major climate change studies (but see Sentinella *et al.*, 2020). This geographical bias is problematic if tropical systems operate qualitatively or quantitatively differently from temperate ones, potentially undermining the conclusions that can be drawn from meta-analyses (Culumber *et al.*, 2019).

Plant seeds are characterized by numerous traits that play a role in ecological functions such as dispersal, persistence, germination timing and establishment (Larson and Funk, 2016; Saatkamp *et al.*, 2019), as well as in *ex situ* conservation, restoration success and sustainable development (Miller and Hobbs, 2007; Li and Pritchard, 2009; Vargas, 2011; Ladouceur et al, 2018; Mattana *et al.*, 2022). Although not all large-scale studies on seeds traits show patterns correlating with geographical/climate zones [e.g. physical defence (Chen and Moles, 2018) and persistence (Gioria *et al.*, 2020)], several seed traits are dominated by different values in tropical versus temperate regions, indicating that data predominantly derived from temperate zones cannot be used to draw general/global conclusions or predict outcomes for the tropics. For example, in a study based on 11 481 species × site combinations, Moles *et al.* (2007) found a 320-fold decline in geometric mean seed mass between the equator and 60° latitude, with a sudden seven-fold drop in mean seed mass at the edge of the tropics. The latitudinal gradient in seed mass (including the step at the edge of the tropics) was almost entirely explained by changes in seed dispersal syndrome, vegetation type, net primary productivity and plant growth form (Moles *et al.*, 2007). A global analysis of legumes (216 000 observations of 532 species) also indicated that larger seeded species are concentrated in tropical regions (Rubio de Casas *et al.*, 2017). In addition, the legume case study revealed that seed dormancy was prevalent in temperate, seasonal environments, whilst non-dormant seeds were more common in the tropics. The latter is in accordance with extensive reviews by Baskin and Baskin (2014) and Zhang *et al.* (2022), in which the largest percentage of non-dormant seeds occurs in tropical regions with the highest rainfall and temperatures (as well as some arctic regions). Baskin and Baskin (2014) also indicated that physiological dormancy seems to be less common in seeds of tropical orchids compared to those from temperate zones, possibly due to symbiotic fungi being active all year. With respect to germination, a global review of 301 taxa found that inhibition by light is absent from humid tropical regions and cold (arctic and high latitudes of Northern temperate zone) climates (Carta *et al.*, 2017). Finally, research involving data from 787 species showed that mean seed dispersal distance increased significantly with latitude towards the tropics (60–0°), for several modes of dispersal (Chen *et al.*, 2019), although abiotic dispersal mechanisms were not analysed separately.

In this report we present three traits in addition to seed size, dormancy, light requirements for germination and dispersal for which existing data indicate differences between tropical [Tropic of Cancer (23.4°N) to Tropic of Capricorn [23.4°S)] and temperate zones [Tropic of Cancer to Arctic Circle (66.6°N) and Tropic of Capricorn to Antarctic Circle (66.6°S)]. We argue that when analysing these traits, especially on a global scale, bias towards temperate zones could have negative consequences for macroecological studies, predictive models and plant conservation. The identified seed traits/syndromes are: (1) desiccation sensitivity, (2) relative embryo size and post-dispersal embryo growth, and (3) germination requirements in high-mountain environments.

## CASE EXAMPLE 1: PREDICTING SEED DESICCATION SENSITIVITY

Research on seed desiccation tolerance plays a pivotal role in plant conservation biology and particularly in *ex situ* conservation (Li and Pritchard, 2009; Wyse and Dickie, 2017). Only seeds that tolerate drying to low moisture contents can be stored below 0 °C for long-term conservation through standard seed banking (FAO, 2014; FAO/IPGRI, 2019). These seeds are defined as ‘orthodox’ and tolerate drying to a moisture content of ~7 % (Roberts, 1973). Species with desiccation-sensitive seeds are known as ‘recalcitrant’ (Roberts, 1973), and alternative *ex situ* conservation actions (e.g. cryopreservation) should be applied to them (Li and Pritchard, 2009). Desiccation-sensitive seeds are mainly limited to climax species and/or to habitats where they are not likely to be exposed to drying and/or freezing conditions after dispersal (Tweddle *et al.*, 2003), such as in tropical rainforests. However, seed desiccation sensitivity is a relatively understudied trait, particularly for species from tropical montane forests (Tweddle *et al.*, 2003; Sommerville *et al.*, 2021). We show below how this lack of tropical data could reduce the predictive power of global models.

According to recent global estimates, 8 % of plant species worldwide are likely to produce desiccation-sensitive seeds (Wyse and Dickie, 2017), a value that rises to 33 % when focusing on tree species (Wyse *et al.*, 2018). However, these global estimates and predictions rely on the modelling of available data, such as those in the Seed Information Database (SID; Royal Botanic Gardens Kew, 2021). The original purpose of this dataset was to provide information on species that could be stored using conventional seed banking, thus being biased both taxonomically and geographically towards desiccation-tolerant species (Wyse and Dickie, 2017). Little is known about the seed desiccation responses of many tropical trees (Sacandé *et al.*, 2005). For example, in a review of South Pacific rainforest species, data on desiccation and cold storage tolerance were lacking for more than half of the 1503 genera examined (Sommerville *et al.*, 2018). This paucity of information for tropical and sub-tropical rainforest species hinders their long-term conservation through conventional seed banking (Sommerville *et al.*, 2021).

Hong and Ellis (1996) developed a protocol to evaluate seed storage behaviour, by assessing seed germination before and after drying. However, this approach uses large numbers of seeds, which are not always available. To reduce the number of seeds needed, Pritchard *et al.* (2004) developed a screening approach for species from the family Arecaceae (the ‘100-seed test’), which was then adapted to assess different floras worldwide (e.g. Hamilton *et al.*, 2013; Gold and Hay, 2014; Lan *et al.*, 2014; Mattana *et al.*, 2020; Sommerville *et al.*, 2021). Mattana *et al.* (2020) compared the gathering of new experimental data through an adaptation of the ‘100-seed test’ and seed trait models (Lan *et al.*, 2014), with global predictions of species seed desiccation likelihood (Wyse and Dickie, 2018), in order to assess the seed desiccation tolerance of native trees from the Dominican Republic (a Caribbean island). While in most cases the two approaches led to comparable results for the investigated species, there were some instances in which they diverged (Mattana *et al.*, 2020). For example, for the two palm species *Coccothrinax fragrans* and *Roystonea borinquena*, the predictive models highlighted a likelihood of seed desiccation sensitivity (Wyse and Dickie, 2018). However, seeds of these two species – in accordance also with their seed weight and moisture content at dispersal (Hong and Ellis, 1996; Lan *et al.*, 2014) – showed a desiccation-tolerant response according to experimental results (Mattana *et al.*, 2020). This discrepancy arose because models had relied on family-level predictions, as no data were available at a lower taxonomic rank. Similar findings were reported by Athugala *et al.* (2021), with six out of 28 tropical montane species studied in Sri Lanka showing a different result in experiments to the prediction made by the phylogenetic affiliation model (Wyse and Dickie, 2017). While these examples confirm the value of such predictive models, they also highlight the importance of generating new data on seed desiccation sensitivity, particularly for populations and species from tropical and sub-tropical regions (see De Jr Lima *et al.*, 2014; Lan *et al.*, 2014; Sánchez *et al.*, 2018; Chau *et al.*, 2019; Waiboonya *et al.*, 2019; Mattana *et al.*, 2020; Ititiaty *et al.*, 2020; Athugala *et al.*, 2021; Sommerville *et al.*, 2021), through screening approaches such as the ‘100-seed test’. These kinds of studies are especially needed for biodiversity hotspots in Mesoamerica, West Africa, Madagascar, Sundaland and Indo-Burma (Pritchard *et al.*, 2022). The resulting data not only can support plant conservation and development programmes in those regions, but also improve the performance of available and future predictive models, as also previously highlighted by Wyse and Dickie (2017, 2018). However, a major challenge is the feeding of new data into existing and yet to be developed predictive models, as this involves the collection of data scattered in the literature, standardization of the data, and inclusion in a central comprehensive dataset which should account for intra- as well as interspecific variability.

## CASE EXAMPLE 2: BIAS OF RELATIVE EMBRYO SIZE AND POST-DISPERSAL EMBRYO GROWTH DATA

Nutrient reserves of Angiosperm seeds are stored either in (1) extra-embryonal tissues such as the endosperm or perisperm, which is typical for early branching clades and families such as Poaceae and Apiaceae, or (2) entirely in the embryo, which is common in Fabaceae and Asteraceae (Martin, 1946). Nutrient reserves that are stored in the endosperm or perisperm can be transferred to the growing embryo or seedling at different stages during germination and establishment. Storing nutrient reserves inside or outside the embryo at dispersal has several implications in terms of the seed germination niche. Seeds with all nutrients stored inside the embryo can germinate and establish faster, which is advantageous in dry and open habitats (Vandelook *et al.*, 2012, 2021; Parsons *et al.*, 2014), while endospermic taxa seem to be at an advantage in shaded and moist environments (Hodgson and Mackey, 1986; Vandelook *et al.*, 2012). The link between relative embryo size (i.e. the size of the embryo in relation to the size of the seed) and germination and dormancy has been extensively studied, focusing mainly on seasonal temperate climate habitats (Baskin and Baskin, 2014). We may, however, expect other relationships in less seasonal tropical areas, for which very little is known (with some exceptions, e.g. Muthuthanthirige *et al.*, 2020). For example, a strong positive relationship has been observed for relative embryo size and seed size (Vandelook *et al.*, 2012). In larger seeds, the amount of nutrients that can be stored outside the embryo relative to the size of the embryo is larger compared to that in smaller seeds. However, whilst seeds were found to be larger towards the tropics (Moles *et al.*, 2007), a study on embryo size in the tropics compared to other regions is still to be done and, as shown below, data for the tropics are largely missing and strongly biased.

To demonstrate the bias of data availability towards non-tropical species, we compiled data on embryo length to seed length ratio and embryo to seed surface ratio for 1295 spermatophyte species, growing either mainly in the tropics or outside the tropics. The number of species sampled is in line with the number of species analysed in the most comprehensive studies on embryo size (Forbis *et al.*, 2002; Verdu, 2006). The data were obtained mainly from pictures or drawings available in the main literature sources dealing with the internal morphology of seeds (e.g. Martin, 1946; Watson and Dallwitz, 1994; Royal Botanic Gardens Kew, 2021) and from measurements on living material (Vandelook *et al.*, 2012, 2021). Measurements were taken on transverse sections of ripe seeds, which had maximal embryo surface area. The embryo to seed length ratio was calculated by measuring total embryo length and dividing this by the length of the seed, measured from the inside of the seed coat, along the longest axis. The embryo to seed surface area was calculated by dividing the embryo area by the area of the embryo plus endosperm and perisperm. In the case of curved or coiled embryos, the lengths of different segments were summed. Species were classified into tropical and non-tropical species based on occurrence coordinates for these species that were downloaded from the Global Biodiversity Information Facility (GBIF download DOI:10.15468/dl.mat4zv). Duplicate coordinates within a species were removed prior to calculation, as well as coordinates linked to institutes rather than sampling locations. For each species, using these coordinates, we calculated the percentage of occurrences in the tropics (between the Tropic of Cancer and the Tropic of Capricorn) and outside of the tropics.

**Fig. 2.**
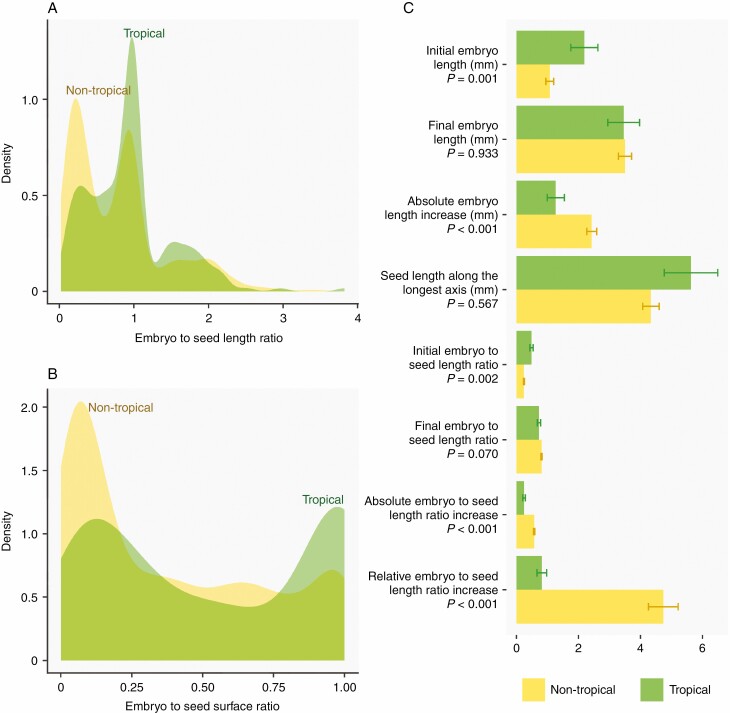
Relative embryo size and post-dispersal embryo growth in tropical vs. non-tropical species. Density plot representing the frequency distribution of (A) embryo to seed length ratio and (B) embryo to seed surface ratio of 1295 spermatophytes either growing mainly in the tropics or outside the tropics. (C) Comparison of several metrics of post-dispersal embryo growth between species either growing mainly in the tropics or outside the tropics: mean initial embryo length (= embryo length in fresh seeds); final embryo length (= embryo length at the moment of germination); absolute embryo length increase (= final embryo length − initial embryo length); seed length along the longest axis; initial embryo to seed length ratio (E : S at the moment of dispersal); final embryo to seed length ratio (E : S at the moment of germination); absolute embryo to seed length ratio increase (= [final embryo length − initial embryo length]/seed length); relative embryo to seed length ratio increase ([final embryo to seed length ratio − initial embryo to seed length ratio]/ initial embryo to seed length ratio). Error bars represent s.e. and *p*-values are the result of a Student’s *t*-test. Seed length was log-transformed prior to testing to meet assumptions of normality.

About 17.5 % of the species for which we obtained records on embryo size had more occurrences in the tropics (a threshold of >50 % occurrences in the tropics was used to classify species as tropical), showing a strong bias in the literature towards non-tropical species. The 226 tropical species represented 80 plant families, which implies that for many tropical plant families (~350 families with a representative in the tropics based on WFO, 2022) we do not have data on embryo size or only from one or two representatives. The 1069 non-tropical species represented 160 plant families (out of ~300 families; WFO, 2022). Data analysis showed that there were clear differences in relative embryo size between tropical and non-tropical species. Embryo to seed length ratio and embryo to seed surface ratio were significantly larger (Student’s *t*-test*: P* < 0.05) in tropical species (mean ± s.e.: 0.93 ± 0.04 and 0.54 ± 0.03, respectively) as compared to non-tropical species (0.78 ± 0.02 and 0.38 ± 0.01, respectively). The frequency distribution of embryo to seed length ratio, expressed as density, reveals three peaks for both tropical and non-tropical species (Fig. 2A). The first peak refers to species with small embryos (ratio < 0.5) and is more pronounced in non-tropical species. A second peak is situated around 1 and mainly consists of species with linear or spatulate embryos that are as long as the seeds themselves. The last peak (ratio > 1) is much lower and consists of species with curved or folded embryos. Two peaks were observed for the embryo to seed surface area (Fig. 2B): one peak for species with small embryos (surface ratio < 0.25), which was higher for non-tropical species, and one peak for species with large embryos (surface ratio > 0.75) that was higher for tropical species.

The large number of species with small embryos in non-tropical species may be partially attributed to a bias towards studying seed internal morphology of many species from a limited number of families and orders that are known to have small embryos, such as Apiaceae (*n* = 226), Ranunculales (*n* = 71) and Asparagales (*n* = 51). In contrast, only three Asteraceae species and no Orchidaceae species, representing the two most diverse plant families, were present in the dataset. Asteraceae are under-studied because they usually have little or no endosperm (Martin, 1946), while the small seeds of Orchidaceae are difficult to work with. The most well-represented plant family in the tropics was Fabaceae (*n* = 50), species of which typically have little endosperm, while only 20 endosperm-abundant Rubiaceae species were included, despite both families being almost equally species-rich in African rainforests (e.g. Sosef *et al.*, 2017). However, for many enigmatic tropical plant families, we currently do not have any data available on relative embryo size.

In addition to relative embryo size, we compiled a dataset on post-dispersal embryo growth inside the seed, also known as morpho(physiological) dormancy (Baskin and Baskin, 2014). To obtain an accurate view of the frequency and distribution of embryo growth across the angiosperms, we performed a literature search focused on manuscripts documenting growth of the embryo. Initial searches on Web of Science and Google Scholar included the keywords ‘morphological dormancy’, ‘morphophysiological dormancy’ and ‘embryo growth’. The reference lists of the retrieved manuscripts were scanned for additional potentially useful studies. Subsequently, we checked all the sources of the species listed as morphologically or morphophysiologically dormant in Baskin and Baskin (2014). The list of manuscripts that was obtained was reduced by keeping only those studies where actual measurements of initial embryo size and embryo size during or after incubation were carried out. Overall, we obtained a list of 150 studies presenting data on embryo growth for 209 species (Supplementary Data File S1). Where possible, we extracted the following data from the studies for each species: initial embryo length (= embryo length in fresh seeds), final embryo length (= embryo length at the moment of germination), seed length along the longest axis, initial embryo to seed length ratio (E : S length ratio at the moment of dispersal), final embryo to seed length ratio (E : S length ratio at the moment of germination), absolute embryo length increase (= final embryo length − initial embryo length), absolute E : S length ratio increase (= [final embryo length − initial embryo length]/seed length) and relative E : S length ratio increase ([final embryo to seed length ratio − initial embryo to seed length ratio]/initial embryo to seed length ratio). Coordinates of the sampling locations were extracted from the respective studies and were used to categorize species as tropical or non-tropical.

Although the tropics include over 60 % of plant diversity (Raven *et al.*, 2020), tropical species represent only 22 % of the data on post-dispersal embryo growth that is available for angiosperms. Data were available for 171 non-tropical species from 32 plant families and 38 tropical species from 18 plant families (Supplementary Data File S1). Most studies on non-tropical species have focused on Apiaceae (*n* = 44) and Ranunculaceae (*n* = 25), two plant families that are particularly species-rich in northern temperate climates (Stevens, 2001). In addition, there is a bias towards studying herbaceous species in non-tropical areas (66 % of the species studied) and towards woody species in the tropics (60 % of the species studied). The studies on tropical species have often focused on multiple species of the same genus, resulting in a very poor representation of tropical diversity. However, our data analysis points to potential differences in post-dispersal embryo growth between tropical and temperate zones, thereby highlighting the need to address the observed biases in order to verify these findings and perform truly global analyses. Initial embryo length and initial embryo to seed length ratio were significantly lower (*P* < 0.01) in non-tropical species compared to tropical species based on *t*-tests (Fig. 2C). In addition, absolute embryo length increase, as well as absolute and relative embryo to seed length ratio increase, was significantly higher (*P* < 0.001) in non-tropical species (Fig. 2C). No significant differences (*P* > 0.05) were observed for seed length, final embryo length or final embryo to seed length ratio (Fig. 2C). Thus, from the data available (Fig. 2C), it seems that embryo growth is limited in tropical species.

According to a literature review by Baskin and Baskin (2014), about 50 % of the studied plant species in tropical rainforests are considered to have non-dormant seeds, while close to 20 % of all tropical species are assigned to the morphological or morphophysiological dormancy class. Such a high percentage of dormant species being classified as morpho(physio)logically dormant is not found in any other region. In temperate forests, morphophysiological dormancy also reaches about 20 % of all studied species, but there is a higher incidence of dormancy overall, as up to 90 % of temperate species have some form of dormancy. This indicates that among dormant seeds in the tropics, morpho(physio)logical dormancy is potentially more frequent, and highlights the need for further study given the bias towards temperate regions and certain families identified earlier. The lack of knowledge on tropical species is also evident from the work of Baskin and Baskin (2014), as for many species the dormancy class was inferred from that of related species.

## CASE EXAMPLE 3.1: GERMINATION REQUIREMENTS IN TEMPERATE VS. TROPICAL HIGH-MOUNTAIN ENVIRONMENTS

The high-mountain or alpine vegetation belt can be found across the planet in mountain areas that are above the maximum elevation at which trees can grow in a given latitude (Körner and Paulsen, 2004; Körner *et al.*, 2011; Testolin *et al.*, 2020). Earth’s high-mountain plant diversity has two peaks: one in the temperate zone, around mountain systems such as the Rocky Mountains, the Alps and the Himalayas; and the other in the tropics, with a hotspot in the South American páramo (Testolin *et al.*, 2021). It is common for high-mountain plant species to reproduce clonally (Körner, 2021), but sexual reproduction and dispersal through seed remains the main strategy to maintain genetic diversity, to colonize suitable new sites and to migrate in response to climatic changes (Walck *et al.*, 2011). The ecophysiological process of seed germination is therefore a key life stage to maintain the diversity of high-mountain plant communities (Poschlod *et al.*, 2013), and it must be timed to occur in the most appropriate period to ensure the subsequent survival of seedlings (Chambers *et al.*, 1990). This timing of germination is achieved by seed dormancy and seed germination responses to environmental cues including moisture, temperature and light (Bewley *et al.*, 2013; Baskin and Baskin, 2014). However, most seed ecophysiological research on dormancy and germination of high-mountain species has focused on temperate areas.

High-mountain habitats are limiting for plant life because of their low temperatures and unstable substrates (Körner, 2021). Other limiting factors are common to temperate high-mountain zones, but not necessarily to tropical ones: marked seasonal cycles of temperature and short growing seasons (Körner, 2021). Compared to mid-latitude mountains, tropical high-mountain ecosystems are relatively aseasonal (considering solar seasons), and records indicate that tropical high-mountain plant species flower throughout the year (Gehrke, 2018). Tropical high-mountain plants are subjected to year-round stress from night-time frost, instead of facing yearly cycles of snow in winter and high temperatures in summer (Körner, 2021). Unfortunately, the research effort dedicated to tropical high-mountain environments has been much less than that focused on temperate mountain areas, although tropical research is now catching up (e.g. Anthelme and Lavergne, 2018; Tovar *et al.*, 2020; Körner, 2021).

Most seed ecophysiological research has focused on environmental cues that are representative of temperate high-mountain areas: for example, the presence of physiological seed dormancy, and the associated need for a prolonged period of overwintering before seeds can respond to germination cues (Shimono and Kudo, 2005; Schwienbacher *et al.*, 2011; Sommerville *et al.*, 2013; Cavieres and Sierra-Almeida, 2018). It is also common for high-mountain seeds – again, studied in temperate areas – to respond to warm germination conditions (Söyrinki, 1938; Bliss, 1958; Amen, 1966; Billings and Mooney, 1968) in what is thought to be a way of restricting germination to summer and avoid early-season frosts.

A recent meta-analysis synthesized available information on seed germination of temperate high-mountain species (Fernández-Pascual *et al.*, 2021), finding that the germination of strict high-mountain species is characterized by: physiological seed dormancy; a strong need for cold stratification or GA_3_ (gibberellic acid) to break dormancy; warm-cued germination; a positive response to alternating temperatures; a positive response to light; and slow and relatively synchronous germination. The meta-analysis concluded that high-mountain plants from the mid-latitudes do not show a unique germination syndrome, but rather a more extreme version of the cold-adapted temperate germination syndrome (i.e. cold stratification + warm germination). Nevertheless, the same study also highlighted the general lack of seed germination studies for tropical high-mountain species (Fernández-Pascual *et al.*, 2021), which may partly be due to such studies not being available in English and/or in the peer-reviewed literature (see case study 3.2 below).

**Fig. 3.**
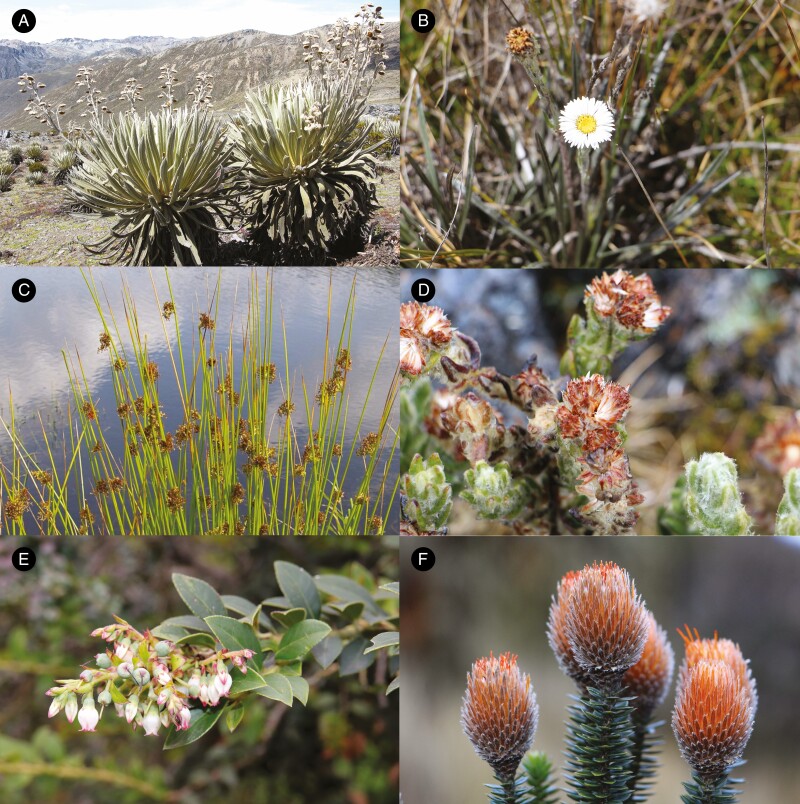
Examples of páramo species. (A) *Espeletia timotensis*; (B) *Oritrophium peruvianum*; (C) *Juncus effusus*; (D) *Monticalia guicanensis*; (E) *Vaccinium meridionale*; (F) *Chuquiraga jussieui*. Credits: Mauricio Diazgranados (A, B, D, F), Christian Fischer (C), David E. Granados (E).

The different selective pressures in tropical high-mountain areas (e.g. lack of thermal seasonality, year-round night frost stress, day/night temperature fluctuations) suggest that the cold-adapted germination syndrome of temperate high-mountain seeds may not be prevalent in the tropical high-mountain flora. Indeed, studies on perennial Asteraceae from the páramos of the Venezuelan Andes showed that their seeds reach high germination percentages over a range of temperatures, and may therefore lack seed dormancy, although germination was lower in the dark (Guariguata and Azocar, 1988; Ulian *et al.*, 2013). Guariguata and Azocar (1988) found that *Espeletia timotensis* (Fig. 3A) seeds germinate to over 75 % between 5 and 19 °C in the light. In addition, seeds of *Oritrophium peruvianum* (Fig. 3B) germinate to ≥60 % when exposed to 24/16 or 26–27 °C in the light, with seedling emergence observed throughout the year and possibly dependent on adequate soil moisture (Ulian *et al.*, 2013). However, other research on páramo species showed germination rates below 40 % at 20/10 °C (12/12-h photoperiod) for several *Espeletia* species with high levels of seed viability, suggesting that they may have a degree of dormancy (Mancipe-Murillo *et al.*, 2018). These examples indicate that more studies are needed on tropical high-mountain species to draw conclusions about the dormancy types and germination syndromes that may be present in these environments.

To date, most assessments of how high-mountain plant regeneration by seed will be affected by climate change have relied on aspects of the temperate–alpine germination syndrome: for example, disruptions in the snow regime breaking the natural cycle of overwintering and dormancy release (Sommerville *et al.*, 2013); or higher autumn temperatures shifting germination from spring to autumn (Mondoni *et al.*, 2012). Assuming that these temperate patterns will hold in the species-rich tropical mountains is very risky: for example, we could expect that the tropical high-mountain seeds of *O. peruvianum* (Ulian *et al.*, 2013), *Puya cryptantha* and *P*. *trianae* (Mora *et al.*, 2007) may be more sensitive to changes in seasonal rainfall patterns. The lack of information on tropical high-mountain seed ecology makes us practically blind when trying to predict the fate of tropical high-mountain floras in a warming climate.

## CASE EXAMPLE 3.2: SEED TRAIT KNOWLEDGE GAPS IN TROPICAL HIGH-MOUNTAIN ECOSYSTEMS: COLOMBIAN PÁRAMO

The páramos are tropical high-mountain ecosystems, typical of the high-elevation mountains mainly located in Colombia, Venezuela and Ecuador and, to lesser extents, in Panamá, Costa Rica and north-east Peru (Luteyn, 1999; Cleef, 2013). They range from the timber line of the Andean Forest, at ~3000 m a.s.l., to the permanent snow line (~4500–5000 m a.s.l.; Cuatrecasas, 1968). These regions are characterized by peculiar and harsh environmental and ecological conditions: high daily temperature fluctuations (with sub-zero temperatures during the night and relatively high temperatures during the day), high radiation (solar and UV), and fast changes in radiation and physiological dryness (Luteyn, 1999). They are listed, as part of the Andes Biodiversity Hotspot, among the global biodiversity hotspots (Myers *et al.*, 2000; Madriñán *et al.*, 2013; Testolin *et al.*, 2020). The páramo hotspot is exceptional in that it is characterized by the world’s fastest plant diversification rates, high endemism and high regional species richness (Madriñán *et al.*, 2013; Testolin *et al.*, 2021).

In addition, páramos provide several ecosystem services such as carbon storage and water supply for cities, agriculture and hydropower (Buytaert *et al.*, 2011). Nevertheless, they are identified as one of the ecosystems most vulnerable to global climate changes (Buytaert *et al.*, 2011), which are predicted to lead to species displacement and local extinction (Ramírez-Villegas *et al.*, 2014; Peyre *et al.*, 2020), with consequent loss of the services provided by this ecosystem (Diazgranados *et al.*, 2021). In addition, they are impacted by anthropogenic disturbances such as grazing (mainly cattle), agriculture (mainly potato), mining, wildfires, cultivation of exotic species and introduction of invasive species (Pérez-Escobar *et al.*, 2018; Rodríguez *et al.*, 2018; Zomer and Ramsay, 2018).

**Fig. 4.**
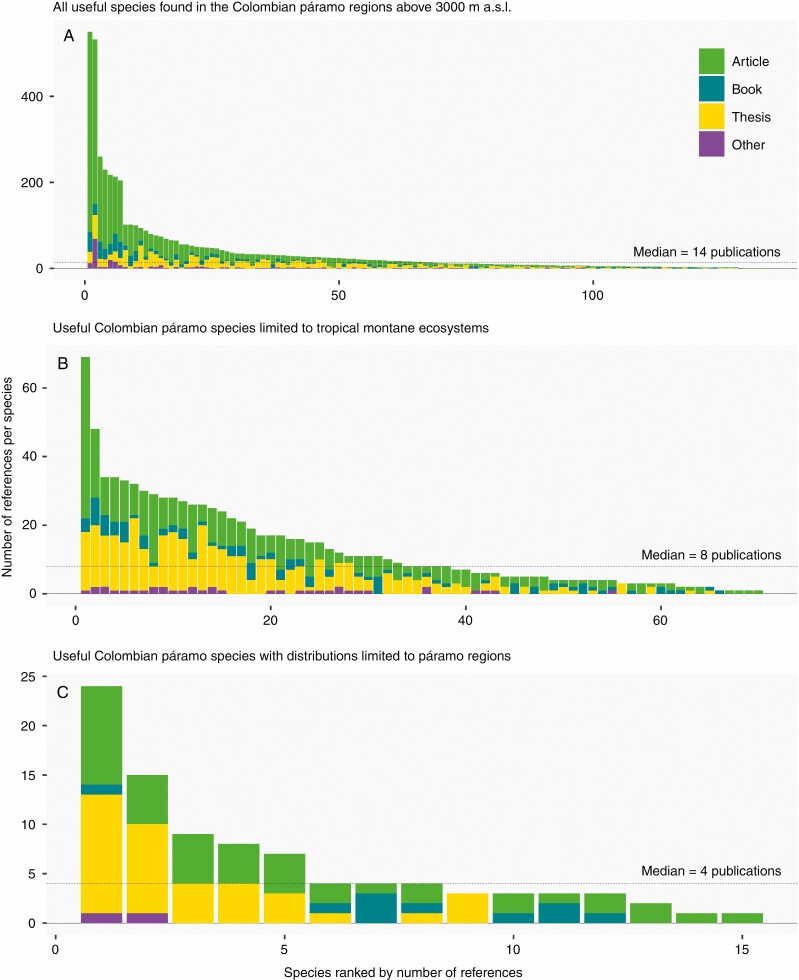
Number of publications on useful páramo species from Colombia. The compiled list corresponds to a sample of 138 species based on Royal Botanic Gardens, Kew – World Checklist of Useful Plants (Diazgranados *et al.*, 2018), the registers of medicinal plants from Colombia (Bernal *et al.*, 2011), and food and medicinal plants according to Instituto de Investigación de Recursos Biológicos Alexander von Humboldt (2014). To evaluate the amount of information on seed traits available in the scientific literature for the useful listed species, a literature search was carried out through Scopus (https://www.scopus.com) and Google Scholar (https://scholar.google.co.uk) using the search string: ‘Generic epithet specific epithet AND *Seed* AND (diaspore OR dispersal OR Embryo* OR germination OR propagation OR dormancy OR mass OR weight OR conservation)’ and the equivalent in Spanish. Retrieved publications were filtered according to the title to exclude duplicated or erroneously included documents. (A) All useful species found in the Colombian páramo regions above 3000 m a.s.l (with any distribution, including cosmopolitan species); (B) useful Colombian páramo species with distributions limited to tropical montane ecosystems; (C) useful Colombian páramo species with distributions limited to páramo regions (‘true páramo species’); dotted lines represent the median publication numbers across species. Literature data up to December 2020.

Despite the evolutionary and ecological importance of and threats facing these tropical high-mountain ecosystems, we show that seed traits of páramo species whose distributions are restricted to tropical montane and páramo regions have been less studied than those of more widely distributed species. For our case study, we quantified the availability of documents reporting on seed traits of species found in the Colombian páramo (see details on the list and methodology applied in Fig. 4). We used the list of Colombian páramo species as a representative sample to quantify the number and types of information sources. Specifically, we focused on ‘useful’ species [i.e. species for which at least one category of uses *sensu*Cook (1995) is reported] because information on plant regeneration from seeds (which is related to their potential cultivation) would be more likely to be available. Literature quantity was analysed according to species distribution (Fig. 4). As expected, peer-reviewed scientific articles were much less frequent than documents from the so-called grey literature (i.e. academic theses, books, reports) (Fig. 4A). In fact, only around 1.8 % of the documents were retrieved from Scopus and this value was reduced as we filtered to páramo species only (for páramo species restricted above 3000 m a.s.l. there were no scientific articles retrieved from Scopus).

As also expected, the number of publications varied greatly among target species. The median was about 14 documents per species, with a maximum of more than 500 documents for the cosmopolitan *Juncus effusus* (Fig. 3C) and none for both *Pentacalia decomposita*, native to Colombia and Venezuela (http://powo.science.kew.org/taxon/urn:lsid:ipni.org:names:909415-1), and *Monticalia guicanensis* (Fig. 3D), endemic to Colombia (http://powo.science.kew.org/taxon/urn:lsid:ipni.org:names:963584-1). Almost 50 % of the species had fewer publications than the median (Fig. 4A). When filtering by species with distributions restricted to tropical montane ecosystems (73 species), the median of relevant documents available per species is reduced to eight (Fig. 4B). *Vaccinium meridionale* (Fig. 3E), a species with edible fruits (Romero Castañeda, 1961), had the maximum number of publications (69 in total). If we account only for species restricted to tropical high-mountain regions above 3000 m a.s.l. (páramo: 15 species), the median decreases to four publications per species (Fig. 4C). *Chuquiraga jussieui* (Fig. 3F), an emblematic species from Bolivia, Colombia, Ecuador and Peru (http://powo.science.kew.org/taxon/urn:lsid:ipni.org:names:194359-1), was the species with the highest number of publications on seed traits (Fig. 4C).

When the information retrieved was filtered by language, the amount of information written in languages different from English was striking; 36.6 % of the documents retrieved were written mostly in Spanish and to a lesser extent in other languages, such as Portuguese. This proportion increased to 69.08 % for species restricted to tropical montane ecosystems, which highlights that most of the available information is generated by local researchers in local languages. Therefore, global analyses of high-mountain seed traits need to include searches in multiple languages of both the grey and peer-reviewed literature in order to cover all currently available information and thereby prevent geographical bias due to language (Konno *et al.*, 2020; Amano *et al.*, 2021; Nuñez and Amano, 2021). This could, for example, be achieved through international collaborations.

In addition, our findings highlight the need for further studies on morphological and functional seed traits of the native páramo flora: particularly for those species typical of these ecosystems, which are restricted to specific habitats, have narrow thermal niches (Cuesta *et al.*, 2020) and have unspecialized seed dispersal (Tovar *et al.*, 2020). The lack of information about the páramo’s (useful) flora makes it difficult to compare high-mountain environments across geographical regions and predict the effects of climate change (see case study 3.1), plan and implement *ex situ* conservation and management programmes, as well as develop regional nature-based solutions (Nesshöver *et al.*, 2017).

## CONCLUSIONS

Our case studies on seed traits with evidence for geographical variation show that the low availability of functional seed trait data from the tropics could have negative consequences for macroecological studies, predictive models and their application in plant conservation. We highlight that the lack of seed desiccation sensitivity data at the population and species level from the tropics may reduce the predictive power of models. In addition, we found a strong bias towards non-tropical species and certain families when analysing existing data on relative embryo size and post-dispersal embryo growth. These traits appear to be quantitatively different between geographical zones, indicating that identified biases need to be corrected in order to perform – and draw accurate conclusions from – global analyses. We also argue that the low number of seed germination studies on tropical high-mountain species prevents both comparisons across geographical regions and predictions related to the effects of anthropogenic climate change in these highly specialized tropical ecosystems. Focusing on the tropical high-mountain páramo environments in Colombia, we show that seed traits of páramo species whose distributions are restricted to tropical montane and páramo regions have been studied less than those of more widely distributed species, with the majority of publications only available in Spanish and/or in the grey literature. This suggests that the geographical bias in data on germination requirements in high-mountain environments could be partly due to language bias.

To prevent the negative consequences of geographical bias on research and predictive models based on global datasets, we suggest: (1) improving inclusion of all existing data by performing multi-lingual searches of both the grey and peer-reviewed literature; (2) generating additional data from the tropics and a wider range of families in both temperate and tropical regions, accounting for intraspecific variability; and (3) improving the availability and accessibility of newly gathered data through their inclusion in seed trait datasets (e.g. Ordóñez-Parra *et al.*, 2022), which could populate comprehensive global databases of seed traits (e.g. SeedArc: http://unioviedo.es/seedarc/). In addition, the availability of seed trait data generated by conservation seed banks could help overcome this geographical bias, as shown for example by the germination data downloaded from the Millennium Seed Bank Partnership Data Warehouse (http://brahmsonline.kew.org/msbp) in Sentinella *et al.* (2020) (see also the methodological approach in Carta *et al.*, 2022). Because reasons for geographical bias include availability of funding and geographical distribution of researchers (Culumber *et al.*, 2019), our suggested strategies could be enabled and supported through additional funding opportunities for tropical plant research, as well as international collaborations.

## Supplementary Material

mcac130_suppl_Supplementary_Data
